# Unveiling the Importance of the Expression of LY6/UPAR Gene Family Members in Urothelial Carcinoma of the Urinary Bladder

**DOI:** 10.3390/biomedicines14061339

**Published:** 2026-06-12

**Authors:** Tuba Dilay Kökenek Ünal, Keziban Korkmaz Bayram, Aida Nurul Barokah, Buse Bayazit Gözüküçük, Umut Inan, Enes Topal, Yusuf Özkul

**Affiliations:** 1 Department of Pathology, Faculty of Medicine, Ankara Yildirim Beyazit University, Ankara 06800, Türkiye; 2 Department of Pathology, Ankara Bilkent City Hospital, Ankara 06800, Türkiye; busebayazit.95@gmail.com; 3 Betül-Ziya Eren Genom and Stem Cell Center (GENKÖK), Erciyes University, Kayseri 38039, Türkiye; 4 Department of Medical Genetics, Faculty of Medicine, Ankara Yildirim Beyazit University, Ankara 06800, Türkiye; drkorkmazbayram@gmail.com; 5 Izmir Biomedicine and Genome Center, Izmir 35340, Türkiye; 6 Department of Translational Medicine, Institute of Health Sciences, Ankara Yildirim Beyazit University, Ankara 06800, Türkiye; biologistaida@gmail.com; 7 Faculty of Medicine, Ankara Yildirim Beyazit University, Ankara 06800, Türkiye; umut067199@outlook.com (U.I.); menest3800@gmail.com (E.T.); 8 Department of Medical Genetics, Faculty of Medicine, Erciyes University, Kayseri 38039, Türkiye; yozkul38@gmail.com

**Keywords:** bladder, cancer stem cell, LY6/uPAR, urothelial carcinoma

## Abstract

**Background/Objectives**: Urothelial carcinomas are the most common tumors of the bladder. There are limited known cancer stem cell markers in these tumors. Ly6/uPAR gene family members are considered to be markers of cancer stem cells and tissue stem cells in mice, but studies on their expression or role in human cancers are limited. In this study, we aimed to investigate the expression of LY6/uPAR gene family members in human urothelial cancers. **Methods**: A total of 84 patients were included in the study. Patients diagnosed with urothelial carcinoma were divided into low-grade noninvasive and high-grade invasive carcinoma groups. Normal urothelial samples were used as a control group. RNA isolation was performed from paraffin blocks, and then cDNA was obtained. *LY6D*, *LY6E*, *LY6H*, *LY6K*, *PSCA*, *LYPD2*, *SLURP1*, *GML*, *GPIHBP1*, and *LYNX1* genes were analyzed by qRT-PCR method. **Results**: We observed significantly higher expression of *LY6E*, *LY6K*, *PSCA*, *GPIHBP1*, and *LYNX1* genes in urothelial carcinomas, but lower expression of *LY6H*, *LYPD2*, and *SLURP1* genes in urothelial cancers compared to the control tissue. Decreased expression of *LY6H*, *PSCA*, *LYPD2*, *SLURP1*, and *GPIHBP1* genes was significantly correlated with poor survival. **Conclusions**: In the present study, the expression of this gene family in bladder cancer was investigated for the first time in the literature. Given their potential prognostic role and possible relevance as therapeutic targets, this study presents preliminary observations that add to the existing literature.

## 1. Introduction

Bladder carcinomas are the fourth most common malignancies among men worldwide [1]. The vast majority of bladder cancers are urothelial carcinomas (UCs) [2]. Smoking is the most common risk factor, and it is responsible for about half of the cases. The other risk factors include exposure to chemical agents, genetic susceptibility, and radiation to the pelvic region [3]. The frequent recurrence of the disease in its early stages, along with the absence of effective treatment options for metastatic and advanced cases, diminishes the overall survival (OS) rate in UCs, highlighting the need for research into new treatment targets [2]. Surgical resection and chemotherapy are well-known and commonly used therapeutic strategies, and immunotherapy has also been tried recently [4,5].

The pathogenesis of UC is well-studied; however, the contribution of cancer stem cells (CSCs) to the disease’s development and progression remains unclear. CSCs—a rare, self-renewing cell population capable of regenerating the tumor—are notoriously therapy-resistant [6]. While standard treatments eradicate the bulk tumor cells, they often fail to target the CSCs that propagate the disease. This failure underpins clinical relapses and metastasis. Therefore, pinpointing these cells, defining new markers for their identification, deciphering their molecular underpinnings, and devising effective CSC-directed therapies are critical research objectives [7].

The lymphocyte antigen 6 (Ly6) gene family is part of the Ly6/uPAR (urokinase-type plasminogen activator receptor) superfamily. Comprising approximately 35 members, this family is implicated in a diverse array of cellular pathways. As evidenced by studies, LY6/uPAR proteins influence a wide variety of processes, including growth, apoptosis, autophagy, immune response, and microenvironment regulation, through their interaction with molecules such as growth factors, nuclear receptors, and miRNAs [8]. Members of this family are surface glycoproteins, expressed across a broad spectrum of cell types, from haematopoietic stem cells to differentiating progenitor cells and fully differentiated cells [9]. The function of the LY6/uPAR gene family, the human homolog of the murine stem cell antigen-1 (SCA-1), a known stem cell marker and oncogene in mice, is not yet fully understood in humans. Given that the murine Ly6 gene family, particularly Sca-1, is widely recognized as a stem cell-associated marker, we hypothesized that its human homologs may similarly be involved in the pathogenesis of urothelial carcinomas. To test this, we aimed to examine the expression levels of ten human LY6/uPAR genes in UC samples. This family includes ten different genes, namely, *LY6D*, *LY6E*, *LY6H*, *LY6K*, *PSCA*, *LYPD2*, *SLURP1*, *GML*, *GPIHBP1*, and *LYNX1* [8,10].

Although a few LY6/uPAR genes (e.g., *LY6D*, *LY6K*, *PSCA*) have already been studied in bladder cancer or pan-cancer contexts, a systematic family-wide expression panel comparison in UCs has not yet been performed. While the literature indicates that the LY6/uPAR family plays a role in the development of various tumors, there is remarkably limited information regarding the comparative expression patterns of multiple family members in UCs. This study aims to investigate the expression levels of LY6/uPAR gene family members in human UCs and to correlate their expression with patients’ clinicopathological parameters, with ultimate goals of evaluating their prognostic value and exploring their potential as therapeutic targets.

## 2. Materials and Methods

Ethical approval for this study was obtained from the Ankara Bilkent City Hospital Ethics Committee (Approval ID: E1-22-2582). All cases were selected from the pathology archive of the hospital. The cohort consisted of 84 subjects, including 60 diagnosed with UC and 24 with normal urothelial epithelium. UC cases were further divided into low-grade noninvasive (NIPUC) and high-grade invasive (IPUC) categories, containing 30 cases each. Non-tumoral (benign) control tissues were selected from cystoscopic biopsy samples of patients without any diagnosis of cancer, for whom the biopsy was performed due to non-oncologic indications. The patients with synchronous or metachronous cancers, or those having neoadjuvant chemotherapy, were excluded from the study.

The demographic data of patients were reported. All hematoxylin and eosin slides were reviewed, and appropriate slides and representative paraffin blocks were selected. The presence of any type of differentiation, variants, or carcinoma in situ foci was noted, but they were not included in the sampling for RNA isolation. Clinicopathological parameters, namely, muscularis propria invasion, lymphovascular invasion, necrosis, differentiation, carcinoma in situ, metastasis, recurrence, disease-free survival (DFS), and OS, were included in the evaluation. Tumor-containing areas with more than 80% tumor cellularity were selected, and macrodissection was performed. Total RNA isolation was performed from the paraffin blocks using the RNeasy FFPE Kit (Qiagen, Hilden, Germany), and the purity and concentration of RNA were measured using a NanoDrop 2000c spectrophotometer (Thermo Fisher Scientific, Waltham, MA, USA). cDNA synthesis was achieved using QuantiTect Reverse Transcription Kit (Qiagen, Hilden, Germany) according to the manufacturer’s protocols. The mRNA expression of *LY6D* (GeneGlobe ID: QT00225596, QuantiTect Primer Assays, Qiagen, Hilden, Germany), *LY6E* (GeneGlobe ID: QT00087521, QuantiTect Primer Assays, Qiagen, Hilden, Germany), *LY6H* (GeneGlobe ID: QT00037681, QuantiTect Primer Assays, Qiagen,Hilden, Germany), *LY6K* (GeneGlobe ID: QT01021524, QuantiTect Primer Assays, Qiagen, Hilden, Germany), *PSCA* (GeneGlobe ID: QT02394329, QuantiTect Primer Assays, Qiagen, Hilden, Germany), *LYPD2* (GeneGlobe ID: QT00068453, QuantiTect Primer Assays, Qiagen, Hilden, Germany), *SLURP1* (GeneGlobe ID: QT00017514, QuantiTect Primer Assays, Qiagen, Hilden, Germany), *GML* (GeneGlobe ID: QT00014945, QuantiTect Primer Assays, Qiagen, Hilden, Germany), *GPIHBP1* (GeneGlobe ID: QT00219296, QuantiTect Primer Assays, Qiagen, Hilden, Germany), and *LYNX1* (GeneGlobe ID: QT01025136, QuantiTect Primer Assays, Qiagen, Hilden, Germany) genes was evaluated by quantitative PCR conducted using StepOnePlus™ Real-Time PCR System (Thermo Fisher Scientific, Waltham, MA, USA) and QuantiTect SYBR Green PCR Kit (Qiagen, Hilden, Germany). The Actin beta gene (GeneGlobe ID: QT01680476, QuantiTect Primer Assays, Qiagen, Hilden, Germany) for mRNA was used as a control. The qRT-PCR analysis was performed using T100 Thermal Cycler PCR (Bio-Rad, Berkeley, CA, USA), the StepOnePlus™ Real-Time PCR System (Thermo Fisher Scientific, Waltham, MA, USA) and the QuantiTect SYBR Green PCR Kit (Qiagen, Hilden, Germany). The analysis was repeated twice, and Ct values were used in a delta–delta Ct (2^−∆∆Ct^) analysis. The comparisons of results with data from public repositories were presented in Supplementary Figures S1 and S2.

The statistical analysis was performed using SPSS 22.0 for Windows (SPSS, Inc.; Chicago, IL, USA). The normal distribution of variables was evaluated using a histogram, a q-q plot, and Shapiro–Wilk’s test. The descriptive statistics were defined as number (n), percentage (%), mean ± standard deviation, or median (IQR). For parametric variables, the independent t-test was performed; for nonparametric variables, the Kruskal–Wallis Test was used to evaluate multiple study groups, and Mann–Whitney U tests were utilized for pairwise comparisons. The Bonferroni correction was applied to adjust significance level to control the overall probability of a Type I error. Potential differences in baseline parameters between groups were assessed using Fisher’s exact test for categorical variables and the appropriate parametric or non-parametric test for continuous variables, as indicated. Survival analyses were performed using the Kaplan–Meier test. To determine the optimal cut-off values for dichotomizing gene expression levels into low- and high-expression groups, receiver operating characteristic (ROC) curve analysis was performed for each gene. The cut-off value that maximized the Youden index (sensitivity + specificity − 1) was selected as the threshold. Patients with expression levels below this cut-off were assigned to the low-expression group, and those with expression levels equal to or above the cut-off were assigned to the high-expression group. A *p* value < 0.05 was considered statistically significant for the study.

## 3. Results

The current study included 84 patients: 69 (82.1%) of patients were male, and 15 (17.9%) were female. In carcinoma cases, 51 (85%) of the patients were male, and 9 (15%) were female. The male-to-female ratio was 5.7:1. The mean age was 69.1 in carcinoma cases. The mean age in carcinoma cases was higher than in normal controls (See Supplementary Table S1). Muscularis propria invasion was observed in only six (10%) of the malignant cases. Recurrence occurred in 23 patients. The mean follow-up period was 30 months, during which 21 patients died. (For general features of the tumoral cases, please see Supplementary Table S2.)

**Figure 1:**
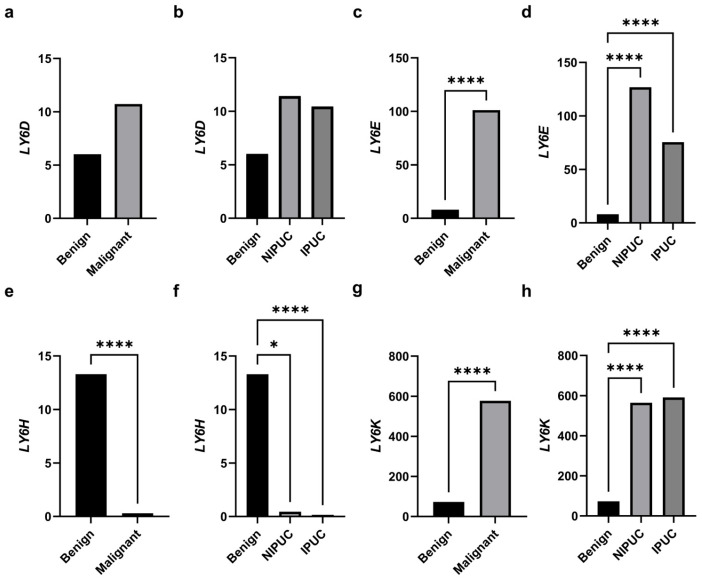
The expression of *LY6D* (**a**), *LY6E* (**c**), *LY6H* (**e**), and *LY6K* (**g**) genes in benign and malignant cases. The expression of *LY6D* (**b**), *LY6E* (**d**), *LY6H* (**f**), and *LY6K* (**h**) in noninvasive and invasive tumors. (*: <0.05; ****: <0.0001) The *y*-axis represents gene expression (2^−∆∆Ct^); the *x*-axis represents groups.

**Figure 2:**
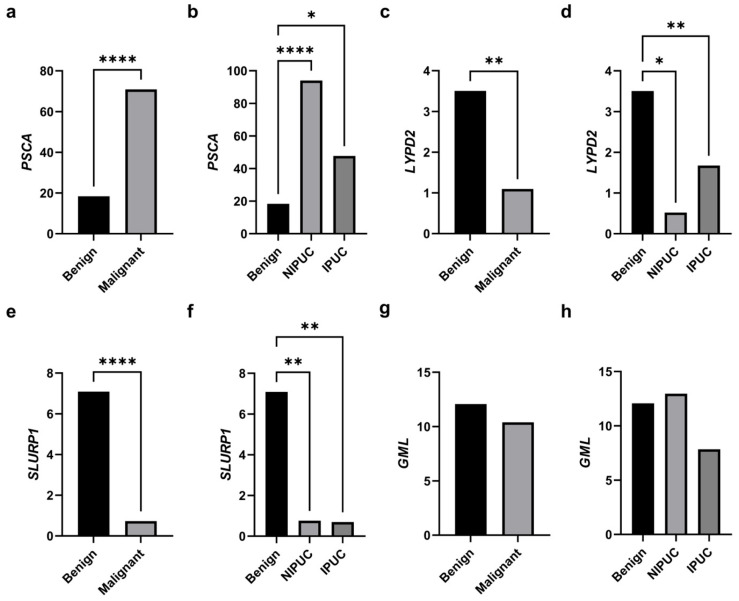
*PSCA* gene expression levels in study groups (**a**,**b**). The expression of the *LYPD2* gene in study groups (**c**,**d**). *SLURP1* expression in study groups (**e**,**f**). *GML* gene expression levels in study groups (**g**,**h**). (*: <0.05; **: <0.01; ****: <0.0001.) The *y*-axis represents gene expression (2^−∆∆Ct^); the *x*-axis represents groups.

**Figure 3:**
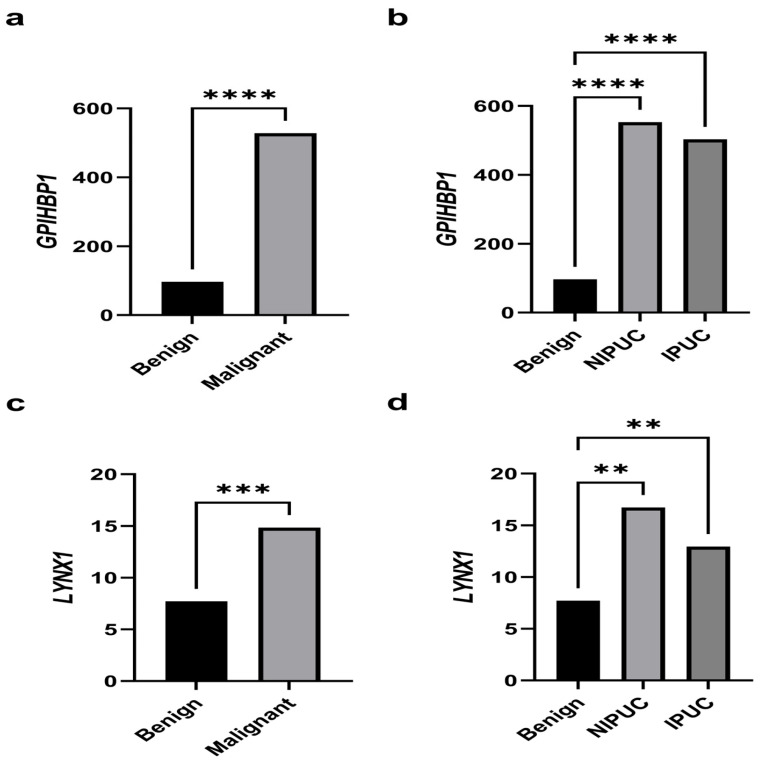
*GPIHBP1* (**a**,**b**) and *LYNX1* (**c**,**d**) gene expression levels in study groups. (**: <0.01; ***: <0.001; ****: <0.0001) The *y*-axis represents gene expression (2^−∆∆Ct^), the *x*-axis represents groups.

The expression of *LY6E*, *LY6H*, *LY6K*, *PSCA*, *LYPD2*, *SLURP1*, *GPIHBP1*, and *LYNX1* genes significantly differed between benign and malignant groups (*p* values are <0.0001, <0.0001, <0.0001, <0.0001, 0.002, 0.0001, <0.0001, 0.0005, respectively) (Table 1 and Figure 1, Figure 2 and Figure 3).

In pairwise comparison, there was a significant difference in the expression of *LY6E*, *LY6H*, *LY6K*, *PSCA*, *LYPD2*, *SLURP1*, *GPIHBP1*, and *LYNX1* between subgoups (*p* values are <0.0001, <0.0001, <0.0001, <0.0001, 0.005, 0.0001, <0.0001, 0.007, respectively) (Table 2 and Table 3). The expression of *LY6E*, *LY6K*, *PSCA*, *GPIHBP1*, and *LYNX1* was significantly higher in the NIPUC and IPUC groups than in the control. On the other hand, the expression of *LY6H*, *LYPD2*, and *SLURP1* was significantly lower in the NIPUC and IPUC groups than in the control. None of the gene expression levels were significantly different between the NIPUC and IPUC groups (Table 2 and Table 3; Figure 1, Figure 2 and Figure 3).

There was no statistically significant relationship between the expression of *LY6D*, *LY6E*, and *LY6K* genes and clinicopathological parameters (See Supplementary Table S3).

**Figure 4:**
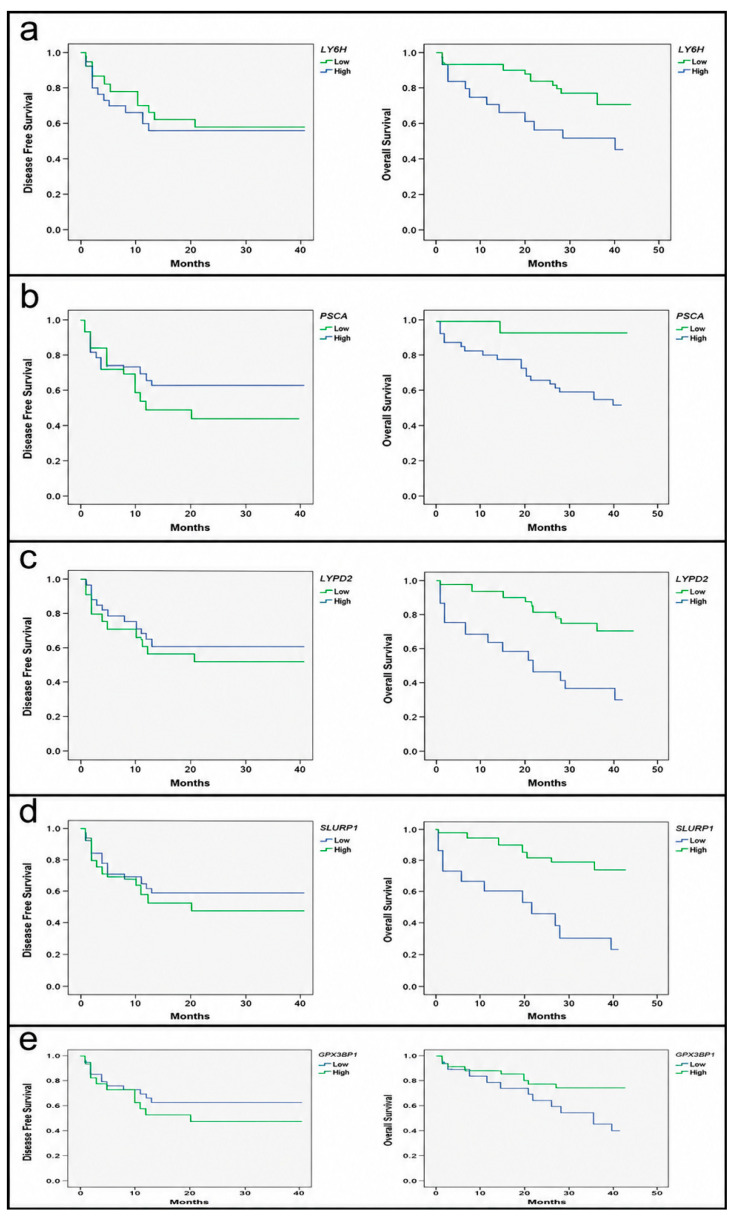
The decreased expression of *LY6H* (**a**), *PSCA* (**b**), *LYPD2* (**c**), *SLURP1* (**d**), and *GPIHBP1* (**e**) was correlated with poor overall survival (*p* values are 0.002, 0.009, 0.001, and 0.016 respectively).

*LY6H* expression was significantly higher in tumors with necrosis (*p* = 0.043). Moreover, the low *LY6H* expression was significantly correlated with poor OS (*p* = 0.002) (Figure 4; see Supplementary Table S4).

PSCA expression was significantly higher in recurrent cases than in nonrecurrent ones (*p* = 0.041), and low expression levels of PSCA were significantly correlated with poor OS (*p* = 0.016) (Figure 4; see Supplementary Table S5).

Although there was no significant relationship between histopathological parameters and the *LYPD2* and *SLURP1* expression levels, the low levels of expression of these genes were significantly correlated with poor OS (*p* = 0.009 and *p* = 0.001, respectively) (Figure 4; see Supplementary Tables S6 and S7).

The *GML* expression was significantly correlated with muscularis propria invasion (*p* = 0.035), despite a nonsignificant difference in its expression levels between tumor and non-tumoral samples (see Supplementary Table S3).

Similarly to the *GML* gene, the expression of the *GPIHBP1* and *LYNX1* genes was significantly higher in muscle-invasive tumors (*p* = 0.037 and *p* = 0.011). However, only the expression level of the *GPIHBP1* gene was significantly correlated with OS (*p* = 0.016) (Figure 4) (See Supplementary Table S8).

In the control group, significant positive correlations were observed among three distinct gene clusters: (1) *LY6D, GML*, and *LYNX1* genes; (2) *LY6H, LYPD2*, and *SLURP1* genes; and (3) *PSCA, GML, GPIHBP1*, and *LYNX1* genes (Table 4). In NIPUCs, we found a significant positive correlation between the expression levels of *LY6E*, *LY6H*, *LY6K*, *PSCA*, *GPIHBP1*, and *LYNX1* genes as well as between the expression levels of *LY6H*, *PSCA*, *LYPD2*, and *GML* genes (Table 5). However, in IPUCs, the correlation pattern changed markedly. Here, *SLURP1* expression showed significant correlation with all other genes. Similarly, *LY6H* expression correlated with all other genes except LY6D. Furthermore, a broad, significant positive correlation was found among the expression levels of *LY6H*, *LY6K*, *PSCA*, *LYPD2*, *SLURP1*, *GML*, *GPIHBP1*, and *LYNX1* genes (Table 6).

## 4. Discussion

This study investigated the expression of genes in the LY6/uPAR gene family and CSC genes in papillary UCs of the bladder. Compared to the control tissue, we found that the expression of *LY6E*, *LY6K*, *PSCA*, *GPIHBP1*, and *LYNX1* genes was significantly increased in UCs, while the expression of *LY6H*, *LYPD2*, and *SLURP1* genes was significantly decreased. We also observed a significant difference in the expression levels of *LY6E*, *LY6H*, *LY6K*, *PSCA*, *LYPD2*, *SLURP1*, *GPIHBP1*, and *LYNX1* in study groups. However, there was no significant difference between NIPUC and IPUC categories. In this study, we also found that decreased expression of *LY6H*, *PSCA*, *LYPD2*, *SLURP1*, and *GPIHBP1* genes was correlated with poor prognosis.

The *LY6/uPAR* gene family encodes glycosylphosphatidylinositol-associated proteins found on the cellular surface. These proteins are expressed on many cell types, particularly immune cells. They have important roles in several physiological and pathological contexts, such as intercellular signaling and immunological response. However, there is growing data about their distinct role in cancer development, invasion, metastasis, and therapy resistance. There is limited data about their expressions in UC and their correlation with clinicopathological parameters.

*LY6D* is highly expressed in squamous cells and urothelial cells and reported to be increased in tumors arising from these cell types [11]. In pancreatic cancers, it was suggested as a useful marker in predicting therapy response and prognosis [12]. *LY6D*, accepted as a stem cell marker, has been reported to inhibit cancer development and invasion, and to reduce the stemness properties of cancer cells, upon its silencing in xenograft mice [13]. *LY6D* was also reported to be correlated with aggressive phenotype, poor prognosis, and poor survival in several cancers, such as squamous cell carcinoma, breast, colorectal, gastric, cervical, and lung cancer [10,14,15]. However, we did not observe any significant correlation between expression level and clinicopathological parameters despite its increased expression in cancers compared to normal urothelium.

*LY6E*, similar to *LY6D*, has been reported to show increased expression in tumoral tissue and to correlate with poor prognosis in many tumors, including ovary, colorectal, stomach, lung, bladder, and brain [10]. Accordingly, we observed significantly increased expression levels in carcinoma compared to the control. However, we observed no relationship with clinicopathological parameters. Preclinical studies showed that *LY6E* promotes cancer growth and migration by inhibiting apoptosis and regulates the Wnt/β-catenin and PTEN/PI3K/Akt/HIF-1 pathways, which are also important in UC development [16,17,18]. Moreover, *LY6E* has been reported to help cancer cells evade the immune system and show resistance to the therapy in breast cancers [19]. A recent study has demonstrated that *LY6E* has a role in T cell activation, and its increased expression in the tumor microenvironment is associated with cytotoxic T cell dismissal in breast cancer and resistance to immunotherapy [20].

There is conflicting data about the expression of *LY6H* in tissues. Limited studies have reported increased and decreased expression levels in similar tumors, such as colorectal, oesophageal, gastric, endometrial, and cervical cancers [10,21]. However, they have reported a higher *LY6H* expression in bladder cancers compared to normal tissue. On the contrary, we found that *LY6H* expression is significantly lower in UC samples and particularly reduced in IPUCs compared to normal controls. One of these limited studies has also mentioned differential expression of *LY6H* in early and late stages of the same tumors, such as UC [21]. We preferentially included patients’ initial tumor samples; therefore, the study cohort predominantly comprises early-stage cases. A recent study has reported that the *LY6H* gene was related to stemness properties and associated with poor prognosis. Contrary to this, we could not find any significant correlation between expression levels and histopathological parameters, except for survival. Moreover, *LY6H* is correlated with other immunomodulatory genes and also associated with immunoinfiltration in tumors, but there are limited studies on this topic [21].

*LY6K* has been reported to increase in several tumors, such as the ovarian, colorectal, brain, cervix, pancreas, lung, stomach, breast, and bladder carcinoma [10,22]. Similarly, we observed a significant increase in the *LY6K* levels in UCs. Limited studies also indicate that LY6K promotes cancer cell proliferation and cell cycle, antibodies against this molecule can inhibit tumor growth, and silencing this gene results in inhibition of cancer growth, migration, and invasion [22,23]. Furthermore, a clinical trial has reported that a vaccine against LY6K stimulates an immune response and provides better OS in patients with head-and-neck cancer [24]. It is also reported that the LY6K vaccine effectively reduced the tumor size in gastric cancer patients [25]. Although no significant correlation with clinicopathological parameters was observed in our study, the increased expression of *LY6K* in tumor samples is noteworthy given its therapeutic potential. Accordingly, its role as a drug target warrants further investigation to evaluate its therapeutic value in UCs.

PSCA is reported as a prognostic marker and is correlated with high Gleason score, advanced stage, and metastasis in prostatic adenocarcinoma [26,27]. Although PSCA is also highly expressed in various tumors such as breast, cervix, pancreas, endometrium, lung, kidney, brain, and bladder, it is underexpressed in esophageal, gastric, and nasopharyngeal carcinomas [28,29,30,31]. Similarly to previous studies, we observed higher *PSCA* expression in UCs than in normal urothelium. We also found that invasive UCs have lower *PSCA* expression than noninvasive UCs. Given the significant correlation between decreased *PSCA* expression and invasive UCs, *PSCA* may serve as a prognostic marker. We observed higher *PSCA* expression in recurrent cases, while low *PSCA* expression is associated with poor overall survival. These findings may reflect the distinct biology of low-grade versus high-grade tumors. Recurrence is more common in low-grade, non-muscle-invasive tumors, which generally have better survival outcomes than high-grade, invasive cancers. Since low-grade and high-grade UCs develop through different molecular pathways, *PSCA* may play divergent roles in each context: promoting recurrence in low-grade tumors while being downregulated in aggressive, high-grade tumors where overall survival is poor. These findings are hypothesis-generating and require validation in larger, grade-stratified cohorts.

LYPD2 is poorly characterized; limited studies have reported its upregulation in gallbladder, cervical, and head-and-neck cancers [8,32,33]. In contrast to them, we observed that UCs had lower expression levels of LYPD2, and decreased expression levels are significantly correlated with poor survival for the first time in the literature. Although no correlation was observed with other pathological parameters in our study, the findings of this pioneering study warrant further investigation into the role of LYPD2 in UCs.

SLURP1 is a late marker of epithelial differentiation and has a role in the regulation of nicotinic acetylcholine receptor (nAChR) signaling, similar to LYNX1, PSCA, and LY6H [8]. It has an anti-carcinogenic effect, serving as a selective ligand for the α7 subunit of nicotinic receptors [34]. Cigarette-triggered cancer development and progression have been thought to be related to the activation of this subunit [35]. Supporting this hypothesis, studies have shown that several cancers decrease SLURP1 levels, and high levels of SLURP1 were protective against malignant transformation in epithelial cells [36,37,38]. Similarly to the literature, we observed lower levels of the *SLURP1* gene in UCs than in normal urothelium. In addition to that, we also reported the decreased SLURP1 levels were correlated with poor OS. Recent studies have reported that synthetic peptides mimicking SLURP1 are effective against squamous cell carcinoma in mouse models [39].

SLURP1 is a good candidate for further investigation in UCs because of several reasons: First of all, it has a role in the regulation of the nAChR signaling pathway, and smoking is the most important etiologic factor in UC and is responsible for half of the cases [3]. Second, the SLURP1 is a detectable marker in body fluids such as sweat, tears, saliva, and urine [40], and urine cytology is the most convenient method for screening and follow-ups in UCs. Lastly, given its protective role in carcinogenesis, this molecule may serve as a promising drug target.

GML plays a key role in the growth of cells with inhibited wild-type p53, as its promoter contains a p53-binding site. Studies on the *GML* gene are limited to cell lines. Differential expression levels of GML were observed among distinct colorectal cancer cell lines, and high GML expression in certain cells increased their sensitivity to anticancer drugs [41]. Similarly, GML has been reported to sensitize cancer cells to chemotherapeutic agents by inducing apoptosis in esophageal and lung cancer cell lines [42,43]. p53 mutations, with which GML interacts, are known to play an important role in the development of IPUCs and in the progression of NIPUCs [44]. Accordingly, we expected that GML expression would vary in UCs. Although our study revealed no significant difference in GML expression between noninvasive and invasive cancers, to the best of our knowledge, this is the first and only study to examine GML expression in human tissues.

Studies regarding the expression of the *GPIHBP1* gene and/or protein in cancer tissues and its association with cancer development and progression are limited. Cancer cells are known to reprogram their cellular metabolism. The GPIHBP1 gene plays a role in intracellular lipolytic processes and triglyceride metabolism [45]. Therefore, it is plausible that GPIHBP1 is involved in cancer development. Indeed, in colorectal carcinomas, GPIHBP1 expression, involved in alterations in fatty acid metabolism, decreases in early stages but increases in advanced diseases, and its suppression at advanced stages inhibits cancer progression [46]. Furthermore, the same study demonstrated that stromal GPIHBP1 expression increased with disease progression, and cases with high expression exhibited a more pronounced immunological response in the tumor microenvironment [46]. In another study investigating transcriptomic profiling of breast cancer, *GPIHBP1* was identified as one of the few genes that were significantly downregulated among hundreds of genes analyzed [47].

In the present study, *GPIHBP1* gene expression was significantly upregulated in both NIPUC and IPUC groups compared to the control. Although no significant difference was observed between invasive and noninvasive tumors, expression levels were significantly higher in cases exhibiting muscle invasion. Survival analysis demonstrated that low *GPIHBP1* expression was associated with shorter survival, suggesting its potential utility as a prognostic marker. To the best of our knowledge, no previous study has reported the prognostic value of *GPIHBP1* in the literature.

The *LYNX1* gene, similarly to *SLURP1* and *PSCA*, serves as an endogenous brake within the nAChR signaling pathway and is a key regulator in smoking-induced cancers. Studies have shown that LYNX1 expression is reduced in most lung cancers relative to adjacent normal tissue, with this decrease becoming more pronounced as tumor differentiation worsens [48]. LYNX1 has been reported to induce apoptosis in lung cancer by promoting cell cycle arrest, while gene silencing has been shown to enhance lung cancer growth [48,49]. Moreover, significant LYNX1 expression at both the gene and protein levels has been detected in colon, lung, skin, and breast cancer cell lines [49]. Another study has reported that increased LYNX1 expression was correlated with unfavorable prognostic parameters in ovarian serous cystadenocarcinomas [50]. In the current study, *LYNX1* gene expression was significantly upregulated in UCs compared to normal urothelium. Although no association was observed with many clinicopathological parameters, expression levels were significantly higher in cases exhibiting deep muscle invasion, representing a more advanced disease stage.

The collective dysregulation of the LY6/uPAR gene family in our study suggests a profound functional shift in the molecular landscape of UC, transitioning from a protective epithelial state to an aggressive, pro-tumorigenic phenotype. Rather than acting as isolated markers, these genes appear to function as an integrated biological network. A key finding of our study is the opposing expression patterns of two distinct subgroups: upregulated genes (*LY6E*, *LY6K*, *PSCA*, *GPIHBP1*, *LYNX1*) and downregulated genes (*LY6H*, *LYPD2*, *SLURP1*). We propose that this reciprocal expression pattern reflects a disruption of the normal homeostatic balance within the LY6/uPAR network, tipping the scales toward tumor progression. The significant upregulation of *LY6E*, *LY6K*, and *PSCA* likely reflects a synergistic activation of oncogenic pathways such as PI3K/Akt/HIF-1, MAPK, and Wnt/β-catenin, which are established drivers of UC cell survival and migration [7]. Additionally, the increased expression of *LYNX1* and *GPIHBP1* may further contribute to this pro-tumorigenic milieu. *LYNX1*, a modulator of nAChR signaling, has been implicated in cellular proliferation, while *GPIHBP1*, though primarily known for its role in lipoprotein metabolism, may influence the metabolic adaptations required for aggressive tumor growth. Molecular studies have demonstrated that *LY6E* and *LY6K* contribute to tumor progression through similar pathways, such as enhanced TGF-β signaling, immune evasion, and increased IFN-γ signaling [8]. In our study, we found that these two genes correlated with each other in both NIPUC and IPUCs. Our observation of significant positive correlations among *LY6E*, *LY6K*, and *PSCA* further supports the concept of a coordinated “pro-oncogenic cluster” within this gene family. Additionally, *GML*, *LY6K, PSCA*, and LY6E have been reported to possess immunomodulatory effects [8]. In our study, we observed that *LY6K* exhibited a significant positive correlation with *LY6E* and *PSCA*, but a negative correlation with GML. Conversely, the marked downregulation of SLURP1 and LY6H represents a critical loss of ‘biological brakes’ within the nAChR signaling axis. Furthermore, significant correlations were observed among *SLURP1*, *LYNX1*, *PSCA*, and *LY6H*, genes that act by modulating the nAChR signaling pathway. *SLURP1*, an anti-carcinogenic nAChR ligand, and *LY6H*, another nAChR modulator, normally exert growth-suppressive effects. Their reduced expression may therefore remove constraint on nAChR-mediated proliferation, particularly in the context of smoking-associated UC. In this integrated model, the depletion of *SLURP1*—an anti-carcinogenic ligand—coupled with the increased expression of *LYNX1* and *PSCA* may facilitate smoking-induced malignant transformation and tumor progression. Our findings that these correlations become more pronounced in IPUC cases further underscore a synergistic model where the ‘oncogenic cluster’ promotes an aggressive phenotype, while the simultaneous loss of ‘differentiative’ members like *LYPD2* predicts significantly poorer overall survival. Similarly, the loss of *LYPD2*, whose function remains less characterized but appears associated with differentiated phenotypes, may further enable tumor dedifferentiation. Importantly, these downregulated genes showed significant correlations with each other and with upregulated members such as *PSCA* and *LYNX1*, suggesting that the balance between these opposing subgroups is disrupted in a coordinated manner. These findings indicate that the altered expression pattern of the LY6/uPAR family in UC does not represent random transcriptional changes but rather an integrated, bidirectional network disruption. While further functional validation is required, this molecular signature offers a promising framework for identifying novel prognostic markers and potential therapeutic targets that may act on multiple nodes within this network.

Although this study provides a significant contribution to the literature as the first to evaluate the expression of LY6/uPAR gene family members in UCs, we were unable to demonstrate the potential differences between NIPUC and IPUC groups, which may have distinct molecular pathways. Although not statistically significant, a trend was observed for several genes. Expression levels of *LY6D*, *LY6E*, *LY6K*, *PSCA*, *GML*, and *GPIHBP1* were elevated in the NIPUC group compared to controls, while they decreased again in the IPUC group. Conversely, *LY6H, LYPD2*, and *SLURP1* showed lower expression in the NIPUC group relative to the controls, with a further decline observed in the IPUC group. Several possible explanations may account for the lack of statistical significance despite the observed trends. First, the sample size within each subgroup may have been insufficient to detect modest differences, leading to limited statistical power. Second, substantial inter-individual variability in gene expression levels within each diagnostic group likely increased the standard deviations, thereby masking potential differences. Third, a limitation of this study is the lack of protein-level validation of the prognostic markers. Ly6/uPAR proteins are cell-surface glycoproteins, and previous reports indicate that mRNA levels do not always correlate with protein expression. Consequently, mRNA expression patterns may not fully reflect biologically meaningful changes at the protein level. Immunohistochemistry would have been desirable to confirm protein expression patterns; however, this could not be performed due to financial constraints. Future studies with larger cohort sizes and protein-level validation are warranted to clarify these trends.

Several other limitations should be considered. The sample size is relatively small, and the number of muscle-invasive cases is particularly limited (n = 6), which may restrict the statistical power and generalizability of findings related to invasive tumors. Furthermore, due to the limited number of metastatic cases, the short follow-up period, and the lack of information regarding the treatment protocols and patients’ responses to therapy, we were unable to make a definitive interpretation regarding its potential prognostic significance. Lastly, functional assays were beyond the scope of this study; therefore, the mechanistic roles of the identified genes in cancer stemness, tumor progression, or treatment resistance remain to be established in future investigations.

## 5. Conclusions

In conclusion, the LY6/uPAR gene family offers an important avenue for research toward improving our understanding of cancer biology and the development of novel therapeutic strategies in UCs. Investigations into the regulation and function of these genes will yield deeper insights into the molecular pathogenesis and advanced personalized oncology approaches in the treatment of UCs. 

## Figures and Tables

**Table 1:** The expression of genes in benign and malignant cases.

	Benign (n = 24) Median (IQR)	Malignant (n = 60) Median (IQR)	*p* Value
*LY6D*	6.01685 (0.22825–21.64740)	10.73075 (5.01625–18.44175)	0.381
*LY6E*	0.63000 (0.81650–18.06585)	42.87730 (27.29015–105.08930)7)	<0.0001
*LY6H*	0.81270 (0.76850–5.16052)	0.04090 (0.00925–0.25017)	<0.0001
*LY6K*	1.01580 (0.015375–67.53190)	302.38975 (206.56385–564.47910)	<0.0001
*PSCA*	4.42545 (0.00785–15.31247)	37.55325 (14.06612–78.26017)	<0.0001
*LYPD2*	1.22740 (0.13860–3.65430)	0.14620 (0.02962–0.57167)	0.002
*SLURP1*	1.35445 (0.201875–6.17075)	0.08235 (0.01412–0.52450)	0.0001
*GML*	2.22960 (0.04282–8.29845)	7.93870 (0.12010–15.86292)	0.091
*GPIHBP1*	8.86075 (0.00437–102.19795)	269.37889 (131.61835–549.38895)	<0.0001
*LYNX1*	1.99800 (0.04225–10.83922)	7.98635 (3.96677–16.89932)	0.0005

**Table 2:** Median values of subgroups and *p* values for multiple comparisons.

	Benign (n = 24) Median (IQR)	NIPUC (n = 30) Median (IQR)	IPUC (n = 30) Median (IQR)	*p* Value
*LY6D*	6.01685 (0.022825–21.64740)	11.42170 (3.75615–17.27482)	10.45045 (3.81327–20.77610)	0.661
*LY6E*	0.63000 (0.081650–18.06585)	48.14275 (33.30035–198.80670)	41.59700 (22.29635–78.54557)	<0.0001
*LY6H*	0.81270 (0.076850–5.16052)	0.10510 (0.014625–0.362325)	0.02455 (0.00692–0.12787)	<0.0001
*LY6K*	1.01580 (0.015375–67.53190)	332.95755 (227.096525–623.45320)	289.70720 (174.76537–574.205975)	<0.0001
*PSCA*	4.42545 (0.00785–15.31247)	41.27780 (23.32817–81.64667)	25.59505 (8.73585–75.07352)	<0.0001
*LYPD2*	1.22740 (0.13860–3.65430)	0.21785 (0.05025–0.60465)	0.10925 (0.022650–0.38137)	0.005
*SLURP1*	1.35445 (0.201875–6.17075)	0.13035 (0.01962–0.62322)	0.05310 (0.00672–0.51950)	0.0001
*GML*	2.22960 (0.04282–8.29845)	11.89415 (0.10447–23.25210)	6.21190 (0.13802–12.32525)	0.090
*GPIHBP1*	8.86075 (0.00437–102.19795)	272.22550 (175.73685–632.68190)	259.26250 (107.38390–574.31172)	<0.0001
*LYNX1*	1.99800 (0.04225–10.83922)	7.58375 (4.0435–17.32837)	9.11245 (3.33902–18.41785)	0.007

NIPUC: noninvasive low-grade urothelial carcinoma; IPUC: invasive high-grade urothelial carcinoma.

**Table 3:** The *p* values obtained from pairwise comparison of genes.

	Benign and NIPUC	Benign and IPUC	NIPUC and IPUC
*LY6D*	0.356	0.542	0.813
*LY6E*	<0.0001	<0.0001	1
*LY6H*	0.047	<0.0001	0.145
*LY6K*	<0.0001	<0.0001	1
*PSCA*	<0.0001	0.013	0.176
*LYPD2*	0.046	0.005	1
*SLURP1*	0.004	0.001	1
*GML*	0.053	0.300	0.114
*GPIHBP1*	<0.0001	<0.0001	1
*LYNX1*	0.007	0.009	1

NIPUC: noninvasive low-grade urothelial carcinoma; IPUC: invasive high-grade urothelial carcinoma.

**Table 4:** Correlation of genes in benign urothelium.

	*LY6D*	*LY6E*	*LY6H*	*LY6K*	*PSCA*	*LYPD2*	*SLURP1*	*GML*	*GPIHBP1*
*LY6D*	1.00								
*LY6E*	0.38	1.00							
*LY6H*	−0.16	−0.15	1.00						
*LY6K*	0.11	0.11	−0.02	1.00					
*PSCA*	0.27	−0.15	−0.01	0.02	1.00				
*LYPD2*	−0.12	0.10	0.679 **	0.06	−0.35	1.00			
*SLURP1*	0.00	0.15	0.726 **	0.18	−0.14	0.690 **	1.00		
*GML*	0.521 **	0.19	−0.13	−0.13	0.621 **	−0.39	0.03	1.00	
*GPIHBP1*	0.00	−0.22	0.26	0.07	0.27	−0.06	0.27	0.421 *	1.00
*LYNX1*	0.487 *	0.14	−0.17	0.12	0.20	−0.21	0.02	0.491 *	0.38

* *p* < 0.05. ** *p* < 0.01.

**Table 5:** Correlation of genes in noninvasive urothelial carcinomas.

	*LY6D*	*LY6E*	*LY6H*	*LY6K*	*PSCA*	*LYPD2*	*SLURP1*	*GML*	*GPIHBP1*
*LY6D*	1.000								
*LY6E*	0.120	1.000							
*LY6H*	−0.343	0.386 *	1.000						
*LY6K*	0.070	0.570 **	0.246	1.000					
*PSCA*	0.131	0.531 **	0.411 *	0.415 *	1.000				
*LYPD2*	−0.257	0.228	0.593 **	0.326	0.426 *	1.000			
*SLURP1*	0.004	0.186	0.569 **	0.021	0.466 **	0.530 **	1.000		
*GML*	0.065	−0.161	−0.509 **	0.104	−0.124	−0.313	−0.531 **	1.000	
*GPIHBP1*	0.154	0.644 **	0.277	0.712 **	0.535 **	0.327	0.043	−0.151	1.000
*LYNX1*	0.255	0.552 **	0.332	0.617 **	0.605 **	0.346	0.309	−0.244	0.729 **

* *p* < 0.05, ** *p* < 0.01.

**Table 6:** Correlation of genes in invasive urothelial carcinomas.

	*LY6D*	*LY6E*	*LY6H*	*LY6K*	*PSCA*	*LYPD2*	*SLURP1*	*GML*	*GPIHBP1*
*LY6D*	1.00								
*LY6E*	0.183	1.00							
*LY6H*	−0.496 **	0.317	1.00						
*LY6K*	−0.265	0.442 *	0.700 **	1.00					
*PSCA*	−0.086	0.172	0.427 *	0.691 **	1.00				
*LYPD2*	−0.337	0.071	0.627 **	0.552 **	0.201	1.00			
*SLURP1*	−0.410 *	0.406 *	0.802 **	0.570 **	0.398 *	0.530 **	1.00		
*GML*	0.189	−0.281	−0.705 **	−0.488 **	−0.317	−0.471 **	−0.455 *	1.00	
*GPIHBP1*	−0.152	0.489**	0.547 **	0.881 **	0.753 **	0.349	0.493 **	−0.451 *	1.00
*LYNX1*	0.098	0.576 **	0.414 *	0.609 **	0.528 **	0.168	0.426*	−0.588 **	0.755 **

* *p* < 0.05, ** *p* < 0.01.

## Data Availability

The original contributions presented in the study are included in the article; further inquiries can be directed to the corresponding author.

## Supplementary Materials

The following supporting information can be downloaded at https://www.mdpi.com/article/10.3390/biomedicines14061339/s1, Figure S1: Expression analysis of LY6/UPAR gene family members (LY6D, LY6E, LY6H, LY6K, PSCA, LYPD2, SLURP1, GML, GPIHBP1, and LYNX1) in Bladder Urothelial Carcinoma (BLCA) using the GEPIA2 platform [51]. (a) Multiple gene comparison between TCGA primary tumor samples and matched TCGA normal tissue. (b) Multiple gene comparison between TCGA primary tumor samples and an expanded normal baseline combining TCGA and GTEx healthy tissue data; Figure S2: Box plots showing the relative mRNA expression of LY6/UPAR gene family members in Bladder Urothelial Carcinoma (BLCA) based on The Cancer Genome Atlas (TCGA) dataset, generated via the UALCAN platform [52]. Statistical significance between sample groups was evaluated using an unpaired two-sample t-test. The y-axis represents transcript per million, and the x-axis represents the normal (n = 19) and primary tumor (n = 408) groups. Each plot includes lower quartile (Q1), upper quartile (Q3), minimum, maximum, and median. The descriptive statistics (median; Q1–Q3; min.–max.) for each gene are as follows: (a) LY6D (*p* = 0.0009): normal (50.33; 0.78-66.83; 0.00-223.37), and primary tumor (83.18; 5.99–658.56; 0.00–2492.39); (b) LY6E (*p* < 0.0001): normal (173.58; 90.54–207.78; 66.18–328.47), and primary tumor (344.22; 181.59–647.54; 0.41–1584.38); (c) LY6H (*p* = 0.130): normal (0.68; 0.14–0.91; 0.06–1.79), and primary tumor (0.26; 0.09–0.74; 0.00–2.55); (d) LY6K (*p* < 0.0001): normal (3.36; 0.67-10.72; 0.11-24.19), and primary tumor (15.38; 6.15-27.57; 0.00-65.19), (e) PSCA (*p* = 0.835): normal (366.75; 2.69-532.86; 0.08-1,333.47), and primary tumor (438.01; 31.57–2284.03; 0.05–7177.28); (f) LYPD2 (*p* = 0.824): normal (0.00; 0.00–0.00; 0.00–0.14), and primary tumor (0.08; 0.00–0.34; 0.00–3.24); (g) SLURP1 (*p* = 0.289): normal (0.00; 0.00–0.07; 0.00–0.28), and primary tumor (0.25; 0.00–0.99; 0.00–4.20); (h) GML (N/A); (i) GPIHBP1 (*p* < 0.0001): normal (5.23; 3.24–9.19; 1.34–13.22), and primary tumor (0.33; 0.14-0.66; 0.00–2.12); and (j) LYNX1 (*p* = 0.478): normal (19.10; 9.31–27.84; 3.45–51.38), and primary tumor (3.11; 1.30–6.12; 0.12–27.06). Table S1: Distribution of cases; Table S2: The histopathological features of urothelial carcinomas; Table S3: Clinicopathologic parameters and gene expression levels; Table S4: Number of patients at risk for the LY6H gene for Kaplan Meier analysis; Table S5: Number of patients at risk for the PSCA gene for Kaplan Meier analysis; Table S6: Number of patients at risk for the LYPD2 gene for Kaplan Meier analysis; Table S7: Number of patients at risk for the SLURP1 gene for Kaplan Meier analysis; Table S8: Number of patients at risk for the GPIHBP1 gene for Kaplan Meier analysis. References [51,52] are cited in the supplementary materials.

## Abbreviations

The following abbreviations are used in this manuscript:

UC	Urothelial Carcinoma
OS	Overall Survival
CSC	Cancer stem cell
Ly6/uPAR	Lymphocyte antigen 6/urokinase-type plasminogen activator receptor
IPUC	High-grade invasive urothelial carcinoma
NIPUC	Low-grade noninvasive urothelial carcinoma
DFS	Disease-free survival
nAChR	Nicotinic acetylcholine receptor

## References

[1] Siegel R.L., Kratzer T.B., Giaquinto A.N., Sung H., Jemal A. (2025). Cancer statistics, 2025. CA Cancer J. Clin..

[2] Spiess P.E., Agarwal N., Bangs R., Boorjian S.A., Buyyounouski M.K., Clark P.E., Downs T.M., Efstathiou J.A., Flaig T.W., Friedlander T. (2017). Bladder cancer, version 5.2017: Clinical practice guidelines in oncology. JNCCN J. Natl. Compr. Cancer Netw..

[3] Chang S.S., Bochner B.H., Chou R., Dreicer R., Kamat A.M., Lerner S.P., Lotan Y., Meeks J.J., Michalski J.M., Morgan T.M. (2017). Treatment of Non-Metastatic Muscle-Invasive Bladder Cancer: AUA/ASCO/ASTRO/SUO Guideline. J. Urol..

[4] Hanna K.S. (2019). Updates and novel treatments in urothelial carcinoma. J. Oncol. Pharm. Pract..

[5] Zhang C., Liu S., Zhang J., Lu J., Chen Z., Pan B., Liu C., Huang M., Zhan H., Wang H. (2025). A Multifunctional Fe-EGCG@RSL3 Nanomedicine Synergizes Ferroptosis Induction and Tumor Microenvironment Remodeling for Enhanced Bladder Cancer Immunotherapy. Research.

[6] Lee S.Y., Jeong E.K., Ju M.K., Jeon H.M., Kim M.Y., Kim C.H., Park H.G., Han S.I., Kang H.S. (2017). Induction of metastasis, cancer stem cell phenotype, and oncogenic metabolism in cancer cells by ionizing radiation. Mol. Cancer.

[7] Yu Y., Ramena G.E.R. (2012). The role of cancer stem cells in relapse of solid tumors. Front. Biosci..

[8] Upadhyay G. (2019). Emerging Role of Lymphocyte Antigen-6 Family of Genes in Cancer and Immune Cells. Front. Immunol..

[9] Gumley T.P., McKenzie Ian F.C., Sandrin M.S. (1995). Tissue expression, structure and function of the murine Ly-6 family of molecules. Immunol. Cell Biol..

[10] Upadhyay G. (2019). Emerging Role of Novel Biomarkers of Ly6 Gene Family in Pan Cancer. Adv. Exp. Med. Biol..

[11] Andersson N., Ohlsson J., Wahlin S., Nodin B., Boman K., Lundgren S., Jirström K. (2020). Lymphocyte antigen 6 superfamily member D is a marker of urothelial and squamous differentiation: Implications for risk stratification of bladder cancer. Biomark. Res..

[12] Ren M., Zhang J., Zong R., Sun H. (2024). A Novel Pancreatic Cancer Hypoxia Status Related Gene Signature for Prognosis and Therapeutic Responses. Mol. Biotechnol..

[13] Duan J., Wang Y., Chen Y., Wang Y., Li Q., Liu J., Fu C., Cao C., Cong Z., Su M. (2023). Silencing LY6D Expression Inhibits Colon Cancer in Xenograft Mice and Regulates Colon Cancer Stem Cells’ Proliferation, Stemness, Invasion, and Apoptosis via the MAPK Pathway. Molecules.

[14] Feng J. (2023). Identification and validation of molecular subtypes and a 9-gene risk model for breast cancer. Medicine.

[15] Wang J., Sheng N., Li Y., Fan Y., Nan X., Fu R. (2023). Ly6D facilitates chemoresistance in laryngeal squamous cell carcinoma through miR-509/β-catenin signaling pathway. Am. J. Cancer Res..

[16] Cen K., Zhou J., Yang X., Guo Y., Xiao Y. (2024). Lymphocyte antigen 6 family member E suppresses apoptosis and promotes pancreatic cancer growth and migration via Wnt/β-catenin pathway activation. Sci. Rep..

[17] Hashemi M., Rezaei M., Rezaeiaghdam H., Jamali B., Koohpar Z.K., Tanha M., Bizhanpour A., Asadi S., Jafari A.M., Khosroshahi E.M. (2024). Highlighting function of Wnt signalling in urological cancers: Molecular interactions, therapeutic strategies, and (nano)strategies. Transl. Oncol..

[18] Yeom C.J., Zeng L., Goto Y., Morinibu A., Zhu Y., Shinomiya K., Kobayashi M., Itasaka S., Yoshimura M., Hur C.-G. (2016). LY6E: A conductor of malignant tumor growth through modulation of the PTEN/PI3K/Akt/HIF-1 axis. Oncotarget.

[19] Alhossiny M., Luo L., Frazier W.R., Steiner N., Gusev Y., Kallakury B., Glasgow E., Creswell K., Madhavan S., Kumar R. (2016). Ly6E/K signaling to TGFβ promotes breast cancer progression, immune escape, and drug resistance. Cancer Res..

[20] Lan H., Chen Y., Wu Y., Li L., Zhang R., Chen Y., Zhu Y., Zhang Q. (2024). Ly6E on tumor cells impairs anti-tumor T-cell responses: A novel mechanism of tumor-induced immune exclusion. Cancer Immunol. Immunother..

[21] Qin H., Lu H., Qin C., Huang X., Peng K., Li Y., Lan C., Bi A., Huang Z., Wei Y. (2024). Pan-cancer analysis suggests that LY6H is a potential biomarker of diagnosis, immunoinfiltration, and prognosis. J. Cancer.

[22] Matsuda R., Enokida H., Chiyomaru T., Kikkawa N., Sugimoto T., Kawakami K., Tatarano S., Yoshino H., Toki K., Uchida Y. (2011). LY6K is a novel molecular target in bladder cancer on basis of integrate genome-wide profiling. Br. J. Cancer.

[23] Selvanesan B.C., Varghese S., Andrys-Olek J., Arriaza R.H., Prakash R., Tiwari P.B., Hupalo D., Gusev Y., Patel M.N., Contente S. (2023). Lymphocyte antigen 6K signaling to aurora kinase promotes advancement of the cell cycle and the growth of cancer cells, which is inhibited by LY6K-NSC243928 interaction. Cancer Lett..

[24] Yoshitake Y., Fukuma D., Yuno A., Hirayama M., Nakayama H., Tanaka T., Nagata M., Takamune Y., Kawahara K., Nakagawa Y. (2015). Phase II clinical trial of multiple peptide vaccination for advanced head and neck cancer patients revealed induction of immune responses and improved OS. Clin. Cancer Res..

[25] Ishikawa H., Imano M., Shiraishi O., Yasuda A., Peng Y.-F., Shinkai M., Yasuda T., Imamoto H., Shiozaki H. (2014). Phase i clinical trial of vaccination with LY6K-derived peptide in patients with advanced gastric cancer. Gastric Cancer.

[26] Zhao Z., He J., Kang R., Zhao S., Liu L., Li F. (2016). RNA interference targeting PSCA suppresses primary tumor growth and metastasis formation of human prostate cancer xenografts in SCID mice. Prostate.

[27] Liu L., Li E., Luo L., Zhao S., Li F., Wang J., Luo J., Zhao Z. (2017). PSCA regulates IL-6 expression through p38/NF-κB signaling in prostate cancer. Prostate.

[28] Li K., Huo Q., Minami K., Tamari K., Ogawa K., Na S., Fishel M.L., Li B.-Y., Yokota H. (2023). Exploring the Tumor-Suppressing Potential of PSCA in Pancreatic Ductal Adenocarcinoma. Cancers.

[29] Argani P., Rosty C., Reiter R.E., Wilentz R.E., Murugesan S.R., Leach S.D., Ryu B., Skinner H.G., Goggins M., Jaffee E.M. (2001). Discovery of new markers of cancer through serial analysis of gene expression: Prostate stem cell antigen is overexpressed in pancreatic adenocarcinoma. Cancer Res..

[30] Nayerpour Dizaj T., Doustmihan A., Sadeghzadeh Oskouei B., Akbari M., Jaymand M., Mazloomi M., Jahanban-Esfahlan R. (2024). Significance of PSCA as a novel prognostic marker and therapeutic target for cancer. Cancer Cell Int..

[31] Xu L.P., Qiu H.B., Yuan S.Q., Chen Y.-M., Zhou Z.-W., Chen Y.-B. (2020). Downregulation of PSCA promotes gastric cancer proliferation and is related to poor prognosis. J. Cancer.

[32] Su P.H., Hsu Y.W., Huang R.L., Weng Y.-C., Wang H.-C., Chen Y.-C., Tsai Y.-J., Yuan C.-C., Lai H.-C. (2017). Methylomics of nitroxidative stress on precancerous cells reveals DNA methylation alteration at the transition from in situ to invasive cervical cancer. Oncotarget.

[33] Dixit R., Pandey M., Rajput M., Shukla V.K. (2022). Unravelling of the comparative Transcriptomic Profile of Gallbladder Cancer using mRNA sequencing. Mol. Biol. Rep..

[34] Grando S.A. (2008). Basic and clinical aspects of non-neuronal acetylcholine: Biological and clinical significance of non-canonical ligands of epithelial nicotinic acetylcholine receptors. J. Pharmacol. Sci..

[35] Schuller H.M. (2009). Is cancer triggered by altered signalling of nicotinic acetylcholine receptors?. Nat. Rev. Cancer.

[36] Arredondo J., Chernyavsky A.I., Grando S.A. (2007). Overexpression of SLURP-1 and -2 alleviates the tumorigenic action of tobacco-derived nitrosamine on immortalized oral epithelial cells. Biochem. Pharmacol..

[37] Throm V.M., Männle D., Giese T., Bauer A.S., Gaida M.M., Kopitz J., Bruckner T., Plaschke K., Grekova S.P., Felix K. (2018). Endogenous CHRNA7-ligand SLURP1 as a potential tumor suppressor and anti-nicotinic factor in pancreatic cancer. Oncotarget.

[38] Senevirathne A., Aganja R.P., Hewawaduge C., Lee J.H. (2023). Inflammation-Related Immune-Modulatory SLURP1 Prevents the Proliferation of Human Colon Cancer Cells, and Its Delivery by Salmonella Demonstrates Cross-Species Efficacy against Murine Colon Cancer. Pharmaceutics.

[39] Shlepova O.V., Shulepko M.A., Shipunova V.O., Bychkov M.L., Kukushkin I.D., Chulina I.A., Azev V.N., Shramova E.I., Kazakov V.A., Ismailova A.M. (2023). Selective targeting of α7 nicotinic acetylcholine receptor by synthetic peptide mimicking loop I of human SLURP-1 provides efficient and prolonged therapy of epidermoid carcinoma in vivo. Front. Cell Dev. Biol..

[40] Favre B., Plantard L., Aeschbach L., Brakch N., Christen-Zaech S., de Viragh P.A., Sergeant A., Huber M., Hohl D. (2007). SLURP1 is a late marker of epidermal differentiation and is absent in Mal de Meleda. J. Investig. Dermatol..

[41] Hashimoto Y., Ueda K., Minami K., Watatani M. (2001). The potential clinical value of GML and the p53 gene as a predictor of chemosensitivity for colorectal cancer. Int. J. Clin. Oncol..

[42] Higashiyama M., Miyoshi Y., Kodama K., Yokouchi H., Takami K., Nishijima M., Nakayama T., Kobayashi H., Minamigawa K., Nakamura Y. (2000). p53-Regulated GML gene expression in non-small cell lung cancer: A promising relationship to cisplatin chemosensitivity. Eur. J. Cancer.

[43] Kimura Y., Furuhata T., Shiratsuchi T., Nishimori H., Hirata K., Nakamura Y., Tokino T. (1997). GML sensitizes cancer cells to Taxol by induction of apoptosis. Oncogene.

[44] Zhao M., He X.L., Teng X.D. (2016). Understanding the molecular pathogenesis and prognostics of bladder cancer: An overview. Chin. J. Cancer Res..

[45] Jiang S., Ren Z., Yang Y., Liu Q., Zhou S., Xiao Y. (2023). Biomedicine & Pharmacotherapy The GPIHBP1-LPL complex and its role in plasma triglyceride metabolism : Insights into chylomicronemia. Biomed. Pharmacother..

[46] Gao M., Liao L., Lin Z., Hu X., Jia L., Gong W., Jia X. (2024). Increase in GPIHBP1 expression in advanced stage colorectal cancer indicates poor immune surveillance. Transl. Cancer Res..

[47] Bao Y., Wang L., Shi L., Yun F., Liu X., Chen Y., Chen C., Ren Y., Jia Y. (2019). Transcriptome profiling revealed multiple genes and ECM-receptor interaction pathways that may be associated with breast cancer. Cell Mol. Biol. Lett..

[48] Fu X.W., Song P.F., Spindel E.R. (2015). Role of Lynx1 and related Ly6 proteins as modulators of cholinergic signaling in normal and neoplastic bronchial epithelium. Int. Immunopharmacol..

[49] Bychkov M., Shenkarev Z., Shulepko M., Shlepova O., Kirpichnikov M., Lyukmanova E. (2019). Water-soluble variant of human Lynx1 induces cell cycle arrest and apoptosis in lung cancer cells via modulation of α7 nicotinic acetylcholine receptors. PLoS ONE.

[50] Liu H., Wang A., Ma Y. (2020). Increased expression of LYNX1 in ovarian serous cystadenocarcinoma predicts poor prognosis. Biomed. Res. Int..

[51] Tang Z., Li C., Kang B., Gao G., Li C., Zhang Z. (2017). GEPIA: A Web Server for Cancer and Normal Gene Expression Profiling and Interactive Analyses. Nucleic Acids Res..

[52] Chandrashekar D.S., Karthikeyan S.K., Korla P.K., Patel H., Shovon A.R., Athar M., Netto G.J., Qin Z.S., Kumar S., Manne U. (2022). UALCAN: An Update to the Integrated Cancer Data Analysis Platform. Neoplasia.

