# Supplementary material for: Unveiling the Importance of the Expression of LY6/UPAR Gene Family Members in Urothelial Carcinoma of the Urinary Bladder
Source: Biomedicines. 2026 Jun 12;14(6):1339. doi: 10.3390/biomedicines14061339 (PMC13297188; doi:10.3390/biomedicines14061339)
Supplement: Supplementary file 1 [file biomedicines-14-01339-s001.zip › Supplementary Figure S1_and_S2.pdf]

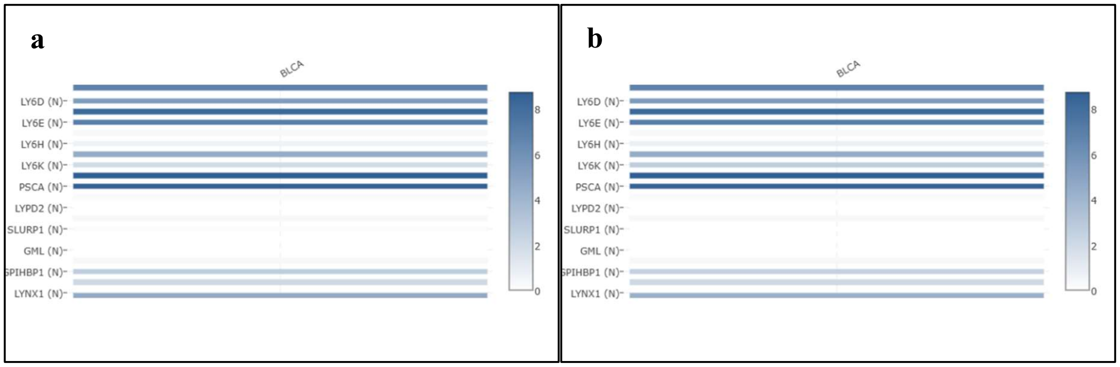

Supplementary Figure S1. Expression analysis of *LY6/UPAR* gene family members (*LY6D, LY6E, LY6H, LY6K, PSCA, LYPD2, SLURP1, GML, GPIHBP1*, and *LYNX1*) in Bladder Urothelial Carcinoma (BLCA) using the GEPIA2 platform [51]. (**a**) Multiple gene comparison between TCGA primary tumor samples and matched TCGA normal tissue. (**b**) Multiple gene comparison between TCGA primary tumor samples and an expanded normal baseline combining TCGA and GTEx healthy tissue data.

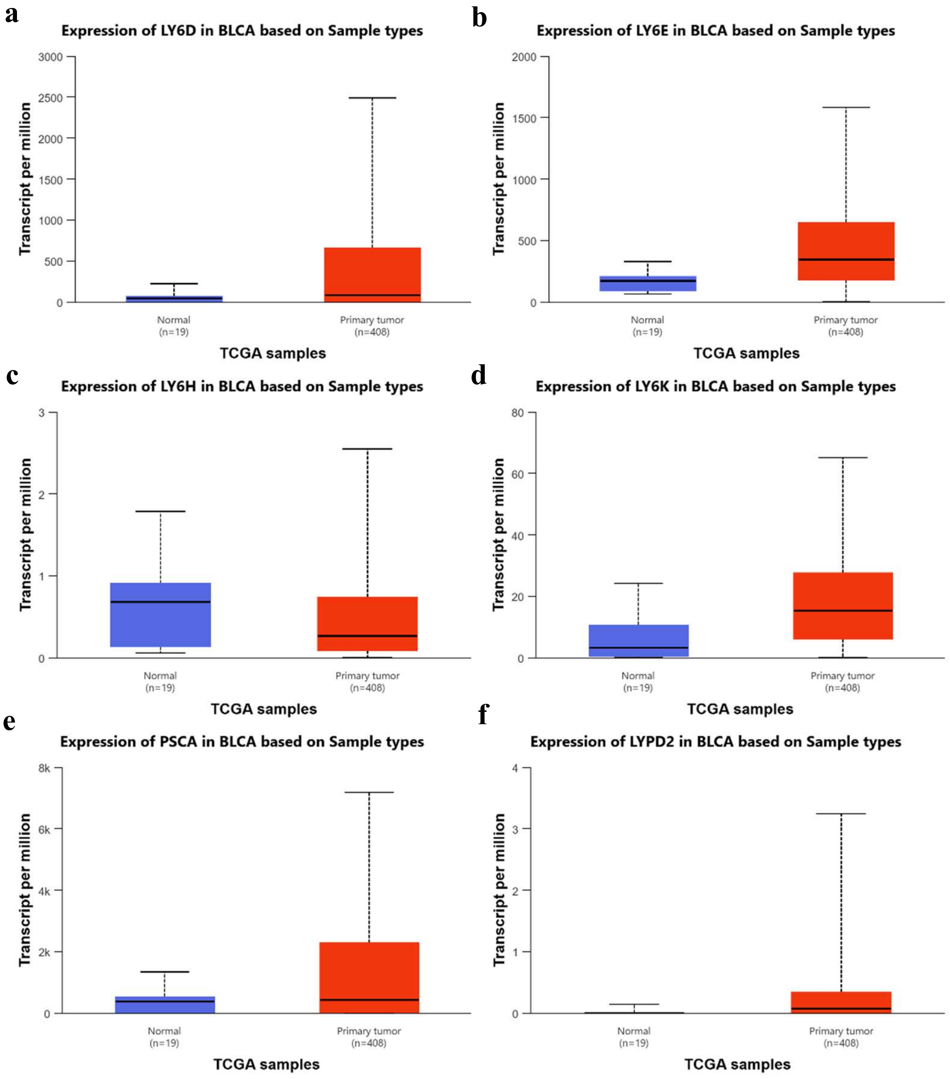
a
Expression of LY6D in BLCA based on Sample types
Transcript per million
3000
2500
2000
1500
1000
500
0
Normal
(n=19)
Primary tumor
(n=408)
TCGA samples
b
Expression of LY6E in BLCA based on Sample types
Transcript per million
2000
1500
1000
500
0
Normal
(n=19)
Primary tumor
(n=408)
TCGA samples
c
Expression of LY6H in BLCA based on Sample types
Transcript per million
3
2
1
0
Normal
(n=19)
Primary tumor
(n=408)
TCGA samples
d
Expression of LY6K in BLCA based on Sample types
Transcript per million
80
60
40
20
0
Normal
(n=19)
Primary tumor
(n=408)
TCGA samples
e
Expression of PSCA in BLCA based on Sample types
Transcript per million
8k
6k
4k
2k
0
Normal
(n=19)
Primary tumor
(n=408)
TCGA samples
f
Expression of LYPD2 in BLCA based on Sample types
Transcript per million
4
3
2
1
0
Normal
(n=19)
Primary tumor
(n=408)
TCGA samples

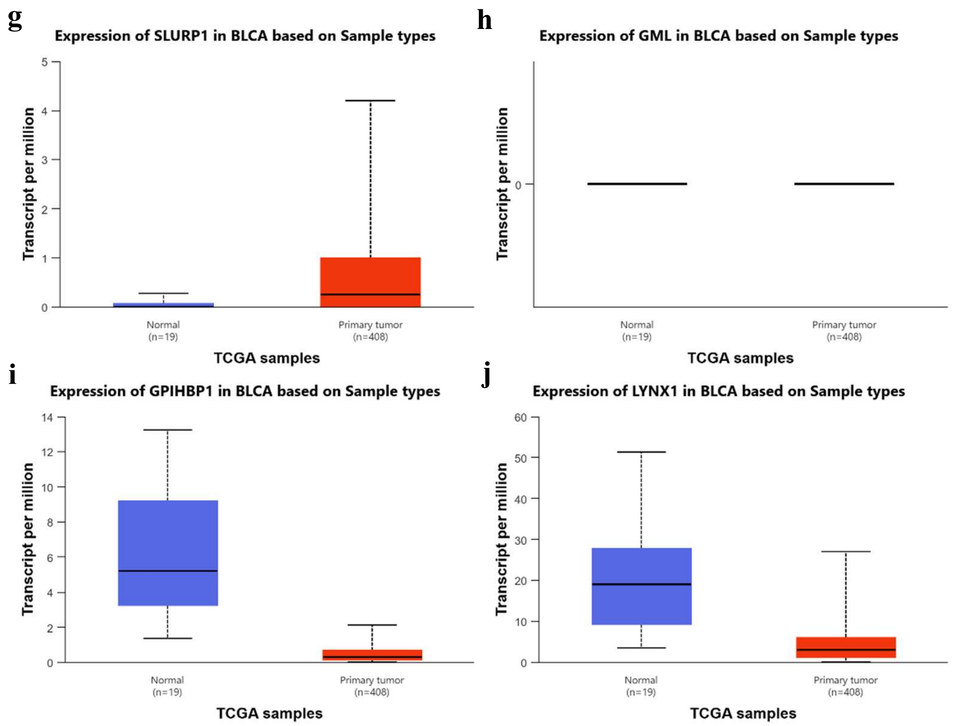

Supplementary Figure S2. Box plots showing the relative mRNA expression of *LY6/UPAR* gene family members in Bladder Urothelial Carcinoma (BLCA) based on The Cancer Genome Atlas (TCGA) dataset, generated via the UALCAN platform [52]. Statistical significance between sample groups was evaluated using an unpaired two-sample t-test. The y-axis represents transcript per million, and the x-axis represents the normal (n=19) and primary tumor (n=408) groups. Each plot includes lower quartile (Q1), upper quartile (Q3), minimum, maximum, and median. The descriptive statistics (median; Q1-Q3; min.-max.) for each gene are as follows: (**a**) *LY6D* (p = 0.0009): normal (50.33; 0.78-66.83; 0.00-223.37), and primary tumor (83.18; 5.99-658.56; 0.00-2,492.39); (**b**) *LY6E* (p < 0.0001): normal (173.58; 90.54-207.78; 66.18-328.47), and primary tumor (344.22; 181.59-647.54; 0.41-1,584.38); (**c**) *LY6H* (p = 0.130): normal (0.68; 0.14-0.91; 0.06-1.79), and primary tumor (0.26; 0.09-0.74; 0.00-2.55); (**d**) *LY6K* (p < 0.0001): normal (3.36; 0.67-10.72; 0.11-24.19), and primary tumor (15.38; 6.15-27.57; 0.00-65.19), (**e**) *PSCA* (p = 0.835): normal (366.75; 2.69-532.86; 0.08-1,333.47), and primary tumor (438.01; 31.57-2,284.03; 0.05-7,177.28); (**f**) *LYPD2* (p = 0.824): normal (0.00; 0.00-0.00; 0.00-0.14), and primary tumor (0.08; 0.00-0.34; 0.00-3.24); (**g**) *SLURP1* (p = 0.289): normal (0.00; 0.00-0.07; 0.00-0.28), and primary tumor (0.25; 0.00-0.99; 0.00-4.20); (**h**) *GML* (N/A); (**i**) *GPIHBP1* (p < 0.0001): normal (5.23; 3.24-9.19; 1.34-13.22), and primary tumor (0.33; 0.14-0.66; 0.00-2.12); and (**j**) *LYNX1* (p =

0.478): normal (19.10; 9.31-27.84; 3.45-51.38), and primary tumor (3.11; 1.30-6.12; 0.12-27.06).

References

1. Tang, Z.; Li, C.; Kang, B.; Gao, G.; Li, C.; Zhang, Z. GEPIA: A Web Server for Cancer and Normal Gene Expression Profiling and Interactive Analyses. *Nucleic Acids Res.* **2017**, *45*, W98–W102, doi:10.1093/NAR/GKX247.
2. Chandrashekar, D.S.; Karthikeyan, S.K.; Korla, P.K.; Patel, H.; Shovon, A.R.; Athar, M.; Netto, G.J.; Qin, Z.S.; Kumar, S.; Manne, U.; et al. UALCAN: An Update to the Integrated Cancer Data Analysis Platform. *Neoplasia* **2022**, *25*, 18, doi:10.1016/J.NEO.2022.01.001.
