# Supplementary material for: Unveiling the Importance of the Expression of LY6/UPAR Gene Family Members in Urothelial Carcinoma of the Urinary Bladder
Source: Biomedicines. 2026 Jun 12;14(6):1339. doi: 10.3390/biomedicines14061339 (PMC13297188; doi:10.3390/biomedicines14061339)
Supplement: Supplementary file 1 [file biomedicines-14-01339-s001.zip › SupplementaryTables_Rev.pdf]

**Supplementary Table S1.** Distribution of cases.

| Diagnosis | Number (%) | Age [min-max] | Gender (%) | |
|---|---|---|---|---|
| | | | Male | Female |
| Control | 24 (28,6% ) | 64.75 [43-84] | 18 (75%) | 6 (25%) |
| NIPUC | 30 (35,7%) | 67 [48-89] | 27 (90%) | 3 (10%) |
| IPUC | 30 (35,7%) | 71,2 [41-90] | 24 (80%) | 6 (20%) |
| Total | 84 (100%) | 67,86 [41-90] | 69 (82,1%) | 15 (17,9%) |

NIPUC: Noninvasive papillary urothelial carcinoma, IPUC: Invasive papillary urothelial carcinoma

**Supplementary Table S2.** The histopathological features of urothelial carcinomas.

| Parameters | Status | Number (%) |
|---|---|---|
| **Gender** | | |
| | Male | 51 (85%) |
| | Female | 9 (15%) |
| **Histopathology** | | |
| | NIPUC | 30(50%) |
| | IPUC | 30 (50%) |
| **Lamina propria invasion** | | |
| | Present | 30 (50%) |
| | Absent | 30 (50%) |
| **Muscularis propria invasion** | | |
| | Present | 6 (10%) |
| | Absent | 54 (90%) |
| **Lymphovascular invasion** | | |
| | Present | 9 (15%) |
| | Absent | 51 (85%) |
| **Necrosis** | | |
| | Present | 8 (13,3%) |
| | Absent | 52 (86,7%) |
| **Differantiation** | | |
| | Present | 14 (23,3%) |
| | Absent | 46 (76,7%) |
| **Carsinoma in situ** | | |
| | Present | 1 (1,7%) |
| | Absent | 59 (98,3%) |
| **Metastasis** | | |
| | Present | 1 (1,7%) |
| | Absent | 59 (98,3%) |
| **Recurrence** | | |
| | Present | 23 (38,3%) |
| | Absent | 37 (61,7%) |
| **Survival** | | |
| | Deceased | 21 (35%) |
| | Alive | 39 (65%) |
| **Follow-up (months)** | 30 [1-41] | |

NIPUC: Noninvasive papillary urothelial carcinoma, IPUC: Invasive papillary urothelial carcinoma

Table S3. Clinicopathologic parameters and gene expression levels.

| | | ***LY6D*** | ***LY6E*** | ***LY6H*** | ***LY6K*** | ***PSCA*** | ***LYPD2*** | ***SLURP1*** | ***GML*** | ***GPIHBP1*** | ***LYNX1*** |
|---|---|---|---|---|---|---|---|---|---|---|---|
| **Muscularis propria invasion** | Present | 25.6300 (4.23847-41.91970) | 115.08220 (44.02475-147.52667) | 0.10650 (0.01137-0.34810) | 332.95755 (227.096525-623.45320) | 89.48560 (32.78590-161.60895) | 0.30355 (0.12287-5.52882) | 0.26650 (0.04272-1.14355) | 1.47360 (0.00507-6.47107) | 934.19245 (382.60000-1987.75947) | 26.39510 (11.03937-37.32717) |
| | Absent | 10.45045 (3.75615-16.89825) | 41.59700 (26.19270-79.05100) | 0.03670 (0.00897-0.26020) | 289.70720 (174.76537-574.205975) | 32.39415 (12.99145-66.73770) | 0.12155 (0.02927-0.56245) | 0.06740 (0.01287-0.51950) | 8.28495 (0.15970-16.64175) | 242.03840 (125.69352-538.80475) | 7.25275 (3.33902-15.64777) |
| | P values | *0.139* | *0.176* | *0.782* | *0.065* | *0.120* | *0.259* | *0.433* | *0.035** | *0.037** | *0.011** |
| **Lymphovascular invasion** | Present | 11.24000 (0.01375-27.04395) | 56.89220 (31.95380-121.52165) | 0.08790 (0.00950-0.66080) | 589.94835 (358.05105-997.33482) | 42.6740 (6.73840-96.30060) | 0.16160 (0.05215-2.84225) | 0.28420 (0.04580-1.52400) | 5.79570 (0.08037-7.72115) | 458.44910 (138.81810-1374.97250) | 9.59630 (5.38065-26.39510) |
| | Absent | 10.53640 (5.07910-17.02490) | 42.84810 (26.41270-103.40480) | 0.03880 (0.00900-0.24800) | 295.274800 (194.39945-462.97957) | 37.14980 (16.91560-79.85350) | 0.13800 (0.02960-0.55780) | 0.06730 (0.00950-0.46980) | 8.28780 (0.11880-17.36360) | 248.24450 (129.93240-540.30030) | 7.54050 (3.93980-16.71550) |
| | P values | *0.959* | *0.612* | *0.733* | *0.325* | *0.992* | *0.514* | *0.192* | *0.196* | *0.414* | *0.528* |
| **Necrosis** | Present | 6.53300 (1.41620-21.62637) | 87.58065 (24.46752-164.53217) | 0.00760 (0.00170-0.09750) | 438.56880 (186.34105-786.63270) | 43.22830 (8.58557-74.85267) | 0.02355 (0.00882-0.21590) | 0.01850 (0.00440-0.36835) | 8.31285 (4.23060-13.28737) | 453.11010 (152.96070-768.74175) | 16.24280 (4.902175-27.78460) |
| | Absent | 11.08255 (5.01625-18.44175) | 42.72455 (27.29015-84.60080) | 0.05470 (0.01152-0.27880) | 297.68840 (205.73730-536.21190) | 35.14240 (17.22542-79.349325) | 0.17095 (0.04915-0.58140) | 0.11150 (0.02030-0.56577) | 7.34700 (0.07582-16.53145) | 242.03840 (131.61835-549.29537) | 7.70985 (3.51837-15.19497) |
| | P values | *0.543* | *0.307* | *0.043** | *0.879* | *0.845* | *0.048** | *0.333* | *0.896* | *0.421* | *0.373* |
| **Differantiation** | Present | 10.24030 (6.50847-17.84777) | 52.07025 (31.75390-196.85002) | 0.01265 (0.00710-0.22182) | 342.83390 (131.03000-804.41562) | 42.31175 (9.95245-85.60060) | 0.07185 (0.01502-0.57800) | 0.04040 (0.01180-0.28815) | 7.18720 (2.48850-13.00512) | 240.17345 (73.16780-928.06340) | 8.16685 (4.78802-25.81450) |
| | Absent | 11.08255 (0.03830-19.70072) | 42.72455 (25.63817-103.96630) | 0.05470 (0.01212-0.37312) | 302.38960 (206.56385-525.08605) | 35.14240 (15.96577-75.43657) | 0.17095 (0.05025-0.58567) | 0.12958 (0.01287-0.60512) | 7.93870 (0.05545-16.85682) | 270.53015 (134.99025-543.32985) | 7.98635 (3.33902-16.06772) |
| | P values | *0.727* | *0.518* | *0.142* | *0.576* | *0.937* | *0.128* | *0.241* | *0.780* | *0.727* | *0.552* |
| **Carsi noma in** | Present | 31.52830 (31.52830-31.52830) | 38.29700 (38.29700-38.29700) | 0.00310 (0.00310-0.00310) | 264.42735 (185.27992-822.62335) | 57.53170 (57.53170-57.53170) | 0.00720 (0.00720-0.00720) | 0.00650 (0.00650-0.00650) | 8.28780 (8.28780-8.28780) | 676.34600 (676.34600-676.34600) | 12.16880 (12.16880-12.16880) |

| | | | | | | | | | | | |
|---|---|---|---|---|---|---|---|---|---|---|---|
| | Absent | 10.53640 (4.99530-18.10830) | 42.90650 (26.41270-105.65080) | 0.04300 (0.01000-0.25090) | 321.59010 (208.43445-545.634300) | 37.14980 (13.11630-79.85350) | 0.15440 (0.02970-0.57630) | 0.09820 (0.01450-0.52700) | 7.74890 (0.11880-16.12240) | 268.47010 (129.93240-540.30030) | 7.79270 (3.93980-16.96060) |
| | P values | *0.233* | *0.800* | *0.200* | *0.800* | *0.633* | *0.100* | *0.333* | *0.933* | *0.467* | *0.700* |
| **Metastasis** | Present | 9.36040 (9.36040 - 9.36040) | 14.98200 (14.98200-14.98200) | 0.00170 (0.00170-0.00170) | 283.39280 (283.39280-283.39280) | 10.21260 (10.21260-10.21260) | 0.09110 (0.09110-0.09110) | 0.00010 (0.00010-0.00010) | 14.42990 (14.42990-14.42990) | 73.14150 (73.14150-73.14150) | 1.66680 (1.66680-1.66680) |
| | Absent | 10.92510 (4.99530-18.55290) | 42.90650 (29.92250-105.65080) | 0.04300 (0.01000-0.25090) | 306.05760 (205.05760-573.90150) | 37.95670 (16.91560-79.85350) | 0.15440 (0.02960-0.57630) | 0.97200 (0.01450-0.52700) | 7.74890 (0.11880-16.12240) | 270.28600 (136.67620-552.41850) | 8.18001 (4.04770-16.96060) |
| | P values | *0.900* | *0.300* | *0.100* | *0.200* | *0.300* | *0.800* | *0.133* | *0.667* | *0.233* | *0.167* |
| **Recurrence** | Present | 11.58280 (5.07910-30.94370) | 58.24890 (36.10580-180.53815) | 0.08070 (0.01380-0.21310) | 119.31730 (119.31730-119.31730) | 54.27760 (24.22600-93.91500) | 0.22000 (0.10360-1.67690) | 0.13540 (0.01400-0.46980) | 6.09230 (0.06150-18.76530) | 292.36320 (155.05300-844.88890) | 10.15500 (4.04770-29.42800) |
| | Absent | 9.36040 (0.02645-15.41715) | 40.59300 (21.27810-64.30675) | 0.02480 (0.00800-0.26950) | 306.05760 (209.04350-573.90105) | 30.60850 (12.22940-56.97550) | 0.08360 (0.02325-0.50660) | 0.06730 (0.01200-0.55285) | 8.12850 (0.84450-14.92415) | 232.10240 (126.25780-539.17850) | 7.54050 (3.14405-15.68855) |
| | P values | *0.105* | *0.087* | *0.569* | *0.162* | *0.041** | *0.056* | *0.749* | *0.970* | *0.327* | *0.204* |
| **Survival** | Present | 10.9251 (2.82350-21.23580) | 40.14530 (16.75130-58.45150) | 0.14200 (0.00300-0.21800) | 334.22120 (283.39280-885.07720) | 24.22260 (9.51175-50.26110) | 0.05530 (0.01660-0.64910) | 0.01990 (0.00510-0.31180) | 8.28780 (2.24005-15.44310) | 163.10050 (79.52170-453.11010) | 6.94090 (2.44460-11.21345) |
| | Absent | 10.53640 (4.99530-16.84880) | 45.89160 (31.71650-137.39250) | 0.09280 (0.01380-0.28810) | 285.36400 (185.21450-406.30200) | 41.48320 (20.39700-102.84190) | 0.21570 (0.05770-0.57630) | 0.13540 (0.03090-0.68440) | 7.74890 (0.03390-17.36360) | 275.98090 (196.66790-854.31110) | 9.00270 (4.95920-29.20450) |
| | P values | *0.932* | *0.147* | *0.028** | *0.079* | *0.016** | *0.165* | *0.027** | *0.727* | *0.025** | *0.062* |

**Supplementary Table S4.** Number of patients at risk for the *LY6H* gene for Kaplan Meier analysis.

| Time (months): | | 0 | 1 | 6 | 12 | 20 | 30 | 40 | 50 | 60 |
|---|---|---|---|---|---|---|---|---|---|---|
| Cut off: 0.149 | Low | 26 | 25 | 22 | 19 | 14 | 11 | 0 | 0 | 0 |
| | High | 58 | 56 | 54 | 48 | 45 | 35 | 10 | 3 | 0 |

**Supplementary Table S5.** Number of patients at risk for the *PSCA* gene for Kaplan Meier analysis.

| Time (months): | | 0 | 10 | 20 | 30 | 40 |
|---|---|---|---|---|---|---|
| Cut off: 54.277 | Low | 42 | 36 | 27 | 24 | 1 |
| | High | 16 | 15 | 2 | 2 | 2 |

**Supplementary Table S6.** Number of patients at risk for the *LYPD2* gene for Kaplan Meier analysis.

| Time (months): | | 0 | 10 | 20 | 30 |
|---|---|---|---|---|---|
| Cut off: 0.577 | Low | 20 | 11 | 3 | 2 |
| | High | 39 | 30 | 20 | 10 |

**Supplementary Table S7.** Number of patients at risk for the *SLURP1* gene for Kaplan Meier analysis.

| Time (months): | | 0 | 10 | 20 | 30 | 40 |
|---|---|---|---|---|---|---|
| Cut off: 0.215 | Low | 17 | 8 | 4 | 1 | 0 |
| | High | 43 | 33 | 8 | 20 | 1 |

**Supplementary Table S8**. Number of patients at risk for the *GPIHBP1* gene for Kaplan Meier analysis.

| Time (months): | | 0 | 10 | 20 | 30 | 40 |
|---|---|---|---|---|---|---|
| Cut off: 189.705 | Low | 21 | 1222 | 6 | 1 | 0 |
| | High | 38 | 30 | 9 | 4 | 1 |
